# Isolation and Characterization of Cytotoxic, Aggregative *Citrobacter freundii*

**DOI:** 10.1371/journal.pone.0033054

**Published:** 2012-03-21

**Authors:** Li Bai, Shengli Xia, Ruiting Lan, Liyun Liu, Changyun Ye, Yiting Wang, Dong Jin, Zhigang Cui, Huaiqi Jing, Yanwen Xiong, Xuemei Bai, Hui Sun, Jin Zhang, Lei Wang, Jianguo Xu

**Affiliations:** 1 State Key Laboratory for Infectious Disease Prevention and Control, National Institute for Communicable Disease Control and Prevention, Chinese Center for Disease Control and Prevention, Beijing, China; 2 Henan Center for Disease Control and Prevention, Zhengzhou, Henan Province, China; 3 School of Biotechnology and Biomolecular Sciences, University of New South Wales, Sydney, New South Wales, Australia; 4 National Institute of Nutrition and Food Safety, Chinese Center for Disease Control and Prevention, Beijing, China; 5 TEDA School of Biological Sciences and Biotechnology, Nankai University, Tianjin, China; University of Hyderabad, India

## Abstract

*Citrobacter freundii* is an infrequent but established cause of diarrhea in humans. However, little is known of its genetic diversity and potential for virulence. We analyzed 26 isolates, including 12 from human diarrheal patients, 2 from human fecal samples of unknown diarrheal status, and 12 from animals, insects, and other sources. Pulsed field gel electrophoresis using *Xba*I allowed us to divide the 26 isolates into 20 pulse types, while multi-locus sequence typing using 7 housekeeping genes allowed us to divide the 26 isolates into 6 sequence types (STs) with the majority belonging to 4 STs. We analyzed adhesion and cytotoxicity to HEp-2 cells in these 26 strains. All were found to adhere to HEp-2 cells. One strain, CF74, which had been isolated from a goat, showed the strongest aggregative adhesion pattern. Lactate dehydrogenase (LDH) released from HEp-2 cells was evaluated as a measure of cytotoxicity, averaging 7.46%. Strain CF74 induced the highest level of LDH, 24.3%, and caused >50% cell rounding, detachment, and death. We named strain CF74 “cytotoxic and aggregative *C. freundii*.” Genome sequencing of CF74 revealed that it had acquired 7 genomic islands, including 2 fimbriae islands and a type VI secretion system island, all of which are potential virulence factors. Our results show that aggregative adherence and cytotoxicity play an important role in the pathogenesis of *C. freundii*.

## Introduction

Adherence to the gastrointestinal mucosa is a key step in the pathogenesis of nearly all enteric bacterial pathogens. Aggregative adherence has been recognized as one of the important modes of pathogenesis. It was first identified in *Escherichia coli* in the 1980s. Enteroaggregative *E. coli* (EAEC) is defined by its characteristic aggregative adherence to HEp-2 cells in culture. Pathogenesis is believed to begin with adherence to the terminal ileum and colon in an aggregative, stacked-brick-type pattern by means of one of several different hydrophobic aggregative adherence fimbriae. EAEC is increasingly recognized as a significant emerging pathogen in several clinical scenarios, such as pediatric diarrhea in both industrialized and developing countries, persistent diarrhea among human immunodeficiency virus (HIV)-infected patients, and a cause of food-borne outbreaks in the industrialized world [1], [2], [3].

*Citrobacter freundii*, a member of the genus *Citrobacter* within the family Enterobacteriaceae, is classically considered a commensal resident in the intestinal tracts of both humans and animals [4]. It has been shown that some isolates have acquired virulence traits and have caused diarrhea and other infections in humans. *C. freundii* has caused sporadic infections and outbreaks [4], [5], [6]. One of the *C. freundii* outbreaks occurred in a nursery in India in 1979. Seventeen babies were infected and one died [7]. Tschape et al. reported a *C. freundii* outbreak of gastroenteritis in a nursery school and kindergarten in Germany which involved 152 cases. Eight patients developed hemolytic uremic syndrome [5]. Recently, a diarrhea-associated aggregative *C. freundii* strain has been isolated. It has been shown to interact with an EAEC through the putative F pili of the EAEC to enhance biofilm formation [8].

The main virulence factors found in diarrhea-associated *C. freundii* are toxins, including Shiga-like toxins and heat stable toxins [5], [9]. The toxigenicity of *C. freundii* was first proposed by Kauffmann and Moller in the 1940s [10]. There is clear evidence that heat-stable toxins and Shiga-like toxins are present in some *C. freundii* strains. Guarino et al. detected heat stable toxins in 46 *C. freundii* strains and, in a later study, showed an 18 amino acid peptide sequenced from a *C. freundii* isolate to be identical to that of *E. coli* ST Ia [9], [11]. Schmidt et al. detected an Stx-II-like toxin in seven *C. freundii* strains. It was found to share 99.58% and 100% identity to *E. coli* SLT-IIvhc [12]. There may be other enterotoxins present in *C. freundii*. Karasawa et al. cloned and sequenced a cholera toxin B subunit homolog from a *C. freundii* strain isolated from a child with diarrhea in Brazil [13]. The toxin was initially detected using anti-cholera toxin polyclonal antibodies. *C. freundii* biotype 4280, now known as *C. rodentium*, carries a type III secretion system (T3SS) similar to that found in enteropathogenic *Escherichia coli* in humans. It produces attaching and effacing lesions in animals [14].

In this study we analyzed a collection of *C. freundii* strains isolated from human diarrheal patients, human-associated animals and insects, and other sources. We identified one strain that showed aggregative adherence pattern and cytotoxicity to HEp-2 cells. The genome of this strain was sequenced and compared to that of a strain with low adherence and low cytotoxicity to HEp-2 cells. We have named this strain cytotoxic and aggregative *C. freundii*. It shows potential to cause diarrheal disease in humans.

## Results and Discussion

### Genetic diversity and molecular relationship of *C. freundii* isolates obtained from human diarrheal patients

During an enteric pathogen investigation in China's Henan Province, we isolated 26 *C. freundii* strains from 12 diarrheal patients, two human fecal samples of unknown diarrheal status, 11 animals and insects, and one other source associated with patients' households or villages (Table 1). The diarrheal patients harbored no other known enteric bacterial pathogens. Viral causes were not investigated. However, all cases occurred during the summer months, and all patients were adults, rendering it more likely that the cases were caused by bacterial infections. *C. freundii* was deemed the causative agent.

**Table 1:** Strains used in this study and their characteristics.

Strain	Origin^1^	Village^2^	PT^3^	ST^3^	O157^4^	Adhesion^5^	LDH^6^	TeR^7^	T6SS^8^
CF65	goat	C	10	1	+	***	7.9±1.2	-	0
CF66	fly	A	1	2	+	*	6.6±1.8	128	0
CF67	goose	A	1	2	+	*	4.8±0.6	128	0
CF69	chopping board	A	7	1	+	**	7.4±1.1	-	0
CF70	goat	F	13	5	+	**	8.1±0.6	-	3
CF71	fly	A	1	2	+	*	6.3±1.5	128	0
CF72	swine	A	1	2	+	*	7.0±1.7	128	0
CF73	fly	D	3	1	+	**	6.9±1.5	-	0
CF74	goat	D	4	6	+	***	24.3±1.3	-	5
CF75	swine	D	3	1	+	**	8.4±1.1	-	0
CF76	fly	D	15	3	+	**	7.4±1.1	-	3
CF77	chicken	B	5	1	+	**	8.4±0.5	-	0
CF78	patient	A	16	2	+	*	5.6±0.4	64	0
CF79	patient	C	14	3	+	*	6.9±0.6	-	3
CF80	patient	A	2	1	+	*	5.7 ±0.2	-	0
CF81	patient	A	2	1	+	**	8.0±1.2	-	0
CF82	patient	E	13	5	+	*	4.5 ±1.0	-	3
CF83	human	F	18	4	-	*	6.2±1.5	-	0
CF84	patient	A	19	4	+	*	6.4 ±0.9	-	0
CF86	patient	D	12	1	+	**	5.2±0.9	-	0
CF88	patient	A	17	4	+	*	6.6±1.7	-	0
CF89	human	D	20	1	+	**	7.4±1.6	-	0
CF90	patient	B	11	1	+	**	5.8±1.6	-	0
CF92	patient	B	9	3	+	**	7.5±1.8	-	3
CF93	patient	C	6	3	+	*	8.4±0.7	-	3
CF94	patient	A	8	2	+	**	6.3±1.0	-	3

1 Patient: confirmed case of diarrhea; human: unknown diarrhea status.

2 Villages coded with letters A to F from which strains were isolated.

3 PT, pulse type; ST, sequence type.

4 O157 antigen determined by agglutination with O157 antiserum.

5 *: adhesion index of <1; **: 1< adhesion index of <50; ***: adhesion index of >50.

6 LDH (%±SD): the lactate dehydrogenase released from HEp-2 cells.

7 Tellurite resistance: Numbers are the tellurite concentration in µg/ml. -: no growth with tellurite concentration at 2 µg/ml, the lowest tested.

8 Five genes from the T6SS were tested (CF74_0731, CF74_0734, CF74_0737, CF74_0743, and CF74_0745). CF_0734 and CF_0745 were not detected in the strains with 3 genes present.

**Figure 1:**
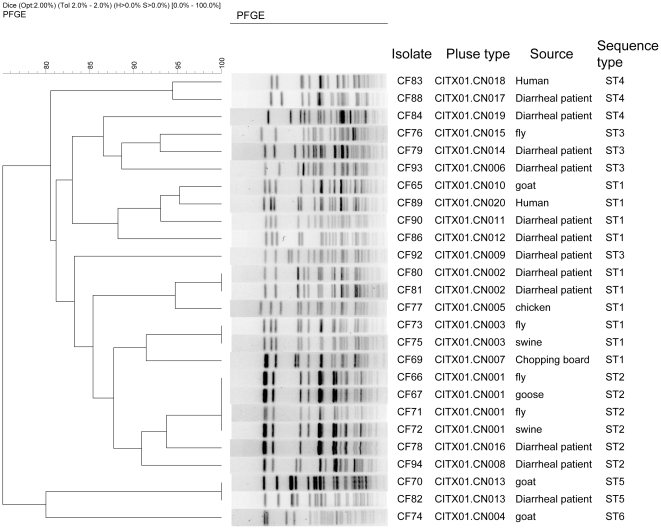
Dendrogram of the 26 *C. freundii* isolates based on PFGE patterns. Shown at the right are PFGE *Xba*I pattern, isolate name, pulse type, source, and sequence type (ST).

The 26 *C. freundii* strains were first analyzed using PFGE with *Xba*I and were differentiated into 20 different PFGE patterns with similarity values ranging from 48.7% to 100%. A UPGMA (unweighted pair group method with arithmetic mean) dendrogram based on Dice confident was constructed (Figure 1). The predominant pulse type was found to be CITX01.CN0001, which has four isolates, all from animal or insect sources. Three types of pulses were found to contain two isolates each, and the remaining pulses each contained a single isolate. The 12 isolates from diarrheal patients were divided into 11 types. Two isolates, CF80 and CF81 from different patients in the same village, were found to share the same pulse type, CITX01.CN0002. Simpson's index of diversity was used to evaluate discriminative power for PFGE. It was found to be 0.972, which is above the power (0.95) recommended as a single typing method for outbreak investigations [15]. On one occasion, human and animal isolates were found to share the same type of pulse, CITX01.CN0013. Also on only one occasion, animal and insect isolates were found to share the same type of pulse, CITX01.CN0003. This suggests that animals may act as a reservoir for human infections and flies may act as a transmission vehicle. Flies are known to transmit bacteria between humans. In a study of *E. coli* O157:H7, it was reported that flies are not only mechanical vectors; O157:H7 has been found multiply inside the flies' mouths and be excreted through fly fecal matter [16].

Because PFGE cannot be used to determine evolutionary relationships, the 26 isolates were further analyzed using multi-locus sequence typing (MLST) of seven housekeeping genes, *aspC*, *clpX*, *fadD*, *mdh*, *arcA*, *dnaG*, and *lysP*, based on Whittam's *E. coli* MLST scheme [17]. The pairwise relative number of differences among the seven genes are shown in Table 2. The most variable gene was found to be *fadD*, which showed maximum and average pairwise relative differences of 5.18% and 2.33%, respectively. The most conserved gene was found to be *arcA*, with maximum and average pairwise relative differences of 0.69% and 0.26%, respectively. However, *aspC* showed the lowest average pairwise relative differences, 0.18%, although it had a higher maximum pairwise relative difference than *arcA* (1.36%). This low level of variation may be due to the narrow representation of the isolates. All except one shared the same O antigen.

**Table 2:** Multi-locus sequence typing genes.

Genes	Primer	Length (bp)	No. alleles	Polymorphic sites (%)	Average % variation (range)
*aspC*	aspCF:5′-GTTTCGTGCCGATGAACGTC-3′	513	3	7 (1)	0.18 (0–1.36)
	aspCR:5′-AAACCCTGGTAAGCGAAGTC-3′				
*clpX*	clpXF:5′-CTGGCGGTCGCGGTATACAA-3′	567	3	15 (3)	0.60 (0–2.29)
	clpXR:5′-GACAACCGGCAGACGACCAA-3′				
*fadD*	fadDF:5′-GCTGCCGCTGTATCACATTT-3′	483	5	32 (7)	2.33 (0–5.18)
	fadDR:5′-GCGCAGGAATCCTTCTTCAT-3′				
*mdh*	mdhF:5′-GTCGATCTGAGCCATATCCCTAC-3′	549	4	12 (2)	0.43 (0–2.00)
	mdhR:5′-TACTGACCGTCGCCTTCAAC-3′				
*arcA*	arcAF:5′-GACAGATGGCGCGGAAATGC-3′	435	4	4 (1)	0.26 (0–0.69)
	arcAR:5′-TCCGGCGTAGATTCGAAATG-3′				
*dnaG*	dnaGF:5′-ACCGCCGATCACATACAACT-3′	444	3	7 (2)	0.21 (0–1.58)
	dnaGR:5′-TGCACCAGCAACCCTATAAG-3′				
*lysP*	lysPF:5′-GCTACGTCGTGAACTGAAGG-3′	477	3	12 (3)	0.9 (0–1.89)
	lysPR:5′-TGTCCCCTGGAAGGAGAAGC-3′				

Each isolate is defined by seven alleles, the combination of which constitutes an allelic profile. Isolates with identical allelic profiles were assigned to the same sequence type (ST). The allelic profiles and STs of the isolates are shown in Table 3. The 26 isolates can be divided into six STs. We further analyzed the six STs using eBURST to identify clonal complexes (CCs)—groups of closely related STs sharing a very recent common ancestor [18]. We used the definition of six out of seven shared alleles for a clonal complex and identified only two CCs. CC1 was found to comprise ST1 and ST3 with 14 isolates in total, and CC2 of ST2 and ST5 with eight isolates. ST1 and ST3 differ in *mdh* by a single base. ST2 and ST5 differ in *fadD* by a single base. All but two of the isolates from diarrhea patients were found to belong to these four STs. Therefore there seems to be a limited number of clones carrying the O157 antigen.

**Table 3:** Multi-locus sequence types of the 26 *C. freundii* isolates.

Sequence				Gene				No.
type	*aspC*	*clpX*	*fadD*	*mdh*	*arcA*	*dnaG*	*lypS*	isolates
ST1	2	1	1	1	2	1	1	10
ST2	1	2	2	3	1	2	2	6
ST3	2	1	1	4	2	1	1	4
ST4	2	1	5	4	3	1	1	3
ST5	1	2	3	3	1	2	2	2
ST6	3	3	4	2	4	3	3	1

**Figure 2:**
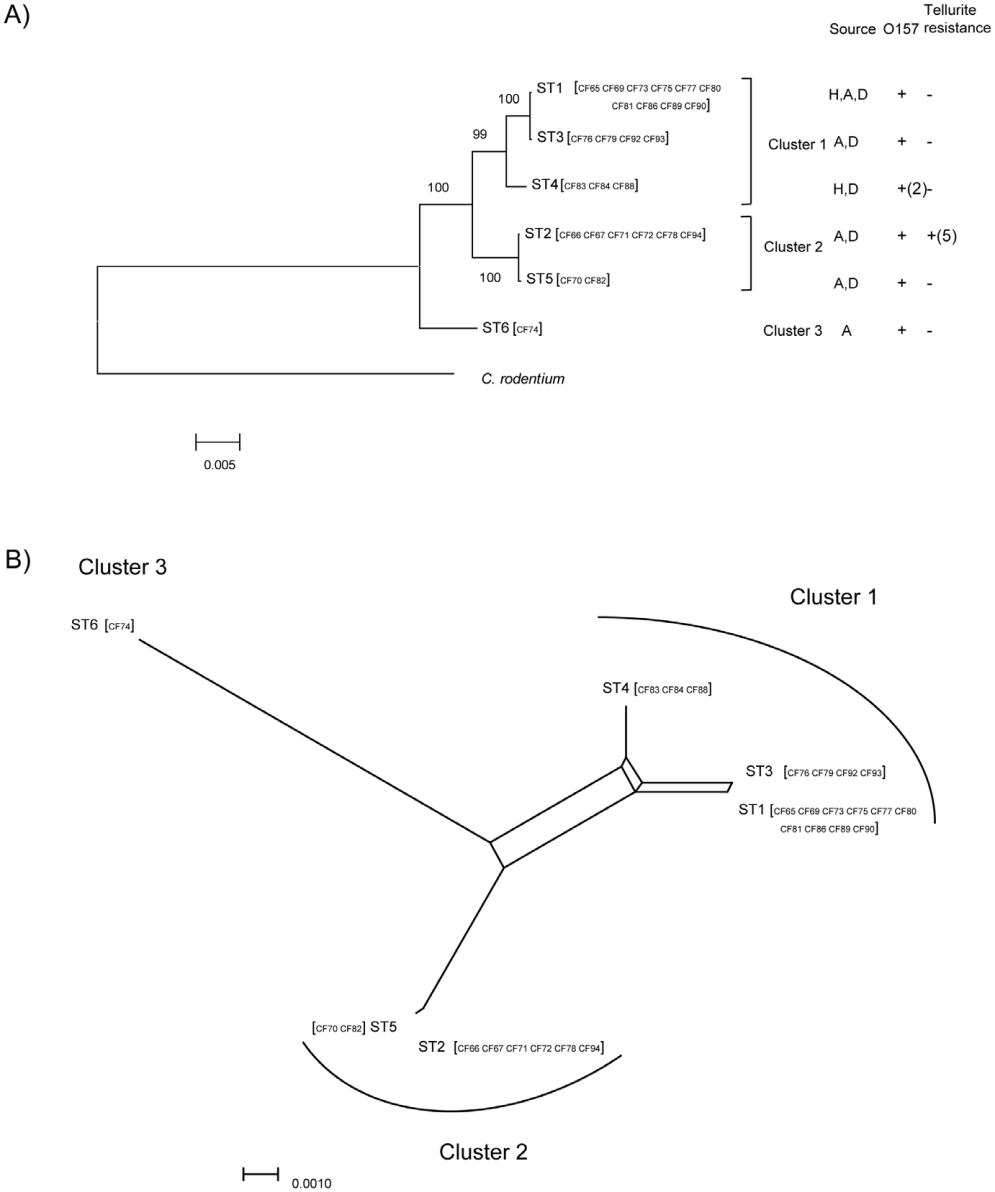
Relationships among the *C. freundii* isolates as indicated by multi-locus sequence typing data. A. Neighbor joining tree showing sequence types (STs). For each ST, sources D, H, and A refer to isolates from diarrheal patients, humans of unknown diarrheal status, and animals or insects. O157 refers to serotyping results for O157 antigen. TR refers to tellurite resistance. B. Neighbor net of the STs. Cluster divisions based on neighbor joining tree is marked on both A and B. After ST in square brackets are isolate names.

We inferred evolutionary relationships of the 26 isolates by constructing a neighbor-joining tree using concatenated sequences of the seven housekeeping genes (Figure 2A). *C. rodentium* was used as the outgroup. The tree can be divided into three clusters with robust bootstrap support of the major divisions. Clusters 1 and 2 were found to be the most closely related. We also constructed a SplitsTree to visualize the effect of recombination which, if present, leads to a network structure. The SplitsTree results showed some network structure (Figure 2B). However, the groupings were the same as those of the neighbor-joining tree. The effect of recombination was small.

The typing data also showed that some PTs and STs could spread over very wide geographic areas. ST1, ST3, ST4, and ST5 were isolated from five, three, two, and two villages, respectively. The villages were all 10 to 60 kilometers apart except for village F, which is about 150 kilometers from the other villages. The two ST5 strains, which are also identical in PFGE pattern, were isolated from two villages 85 kilometers apart.

### HEp-2 cell adherence of *C. freundii* isolates

Adhesion is an essential virulence property of bacterial pathogens. *In vitro* assays have been widely used to assess this property [19]. We tested all 26 isolates for adhesion to HEp-2 cells. Enteropathogenic *E. coli* (EPEC) 2348/69 and EAggEC 042 were used as controls of different types of adhesion and HB101 was used as a negative control. The EPEC strain 2348/69 showed localized adherence, while EAggEC 042 showed aggregative adherence, in keeping with the results of previous studies [20]. All *C. freundii* isolates were found to adhere to HEp-2 cells (Table 1).

Variations in adhesion were observed among the 26 isolates. We categorized the extent of adhesion using the adhesive index [19] (Table 1). Two isolates, CF65 and CF74, both from animal sources, showed the strongest adhesion, with adhesion indexes greater than 50. Twelve isolates showed intermediate adhesion, with adhesion indexes between 1 and 50. The remaining isolates showed little adhesion, with indexes of less than one. These last can be considered non-adhesive because the adhesion index for the control *E. coli* strain HB101 is <1.

**Figure 3:**
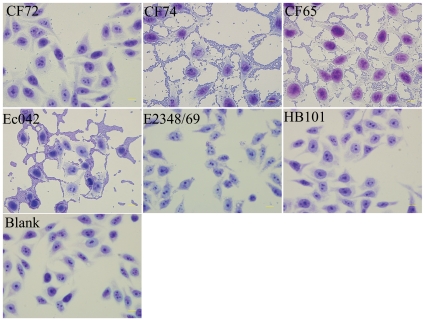
HEp-2 cell adhesion of *C. freundii* isolates. Light micrographs show the adherence patterns displayed by *C. freundii* strains CF72 and CF74 and control bacterial strains as labeled. The prototype EAEC strain 042, EPEC strain 2348/69, and HB101 displayed aggregative adherence, localized adherence, and non-adherence, respectively. Bar: 10 µm.

As shown in Figure 3, the strain with the highest adhesion index, CF74, showed an aggregative adherence pattern similar to that of EAggEC 042 (Figure 3). Adhesion assays were performed with the addition of 1% mannose, which did not abolish adhesion because most strains had adhesion indexes greater than one. We did not evaluate adhesion without mannose. Variations in adhesion due to sensitivity to mannose were therefore not detected.

The variation in adhesion does not seem to be correlated with STs or clusters, as indicated by MLST. The two isolates that showed the highest adhesion, CF74 and CF65, were found in two different clusters. CF74 is located in cluster 2 and CF65 in cluster 1. However, both showed a tendency to associate with particular STs with adhesion ability. All three ST4 isolates and all but one ST2 isolate showed little adhesion, and all except but ST1 isolate showed intermediate or high adhesion.

### HEp-2 cell cytotoxicity of *C. freundii* isolates

**Figure 4:**
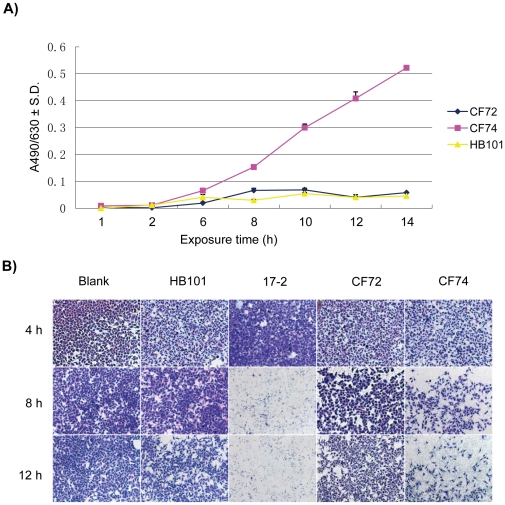
HEp-2 cell cytotoxicity of *C. freundii* isolates. A: Lactate dehydrogenase (LDH) released by HEp-2 cells after growth with *C. freundii* isolates. LDH release from HEp-2 cells after exposure to CF72, CF74, and HB101 at different points in time (hours) was measured using optical density reading at A490/630 (vertical axis). B: Morphological changes of HEp-2 cells after growth with *C. freundii* isolates. Cell rounding, detachment, and death can be seen clearly after 8 h of exposure to *C. freundii* CF74 in comparison to *C. freundii* CF72, *E coli* HB101 and 17-2. Bar: 50 µm.

All isolates were tested for cytopathogenicity to cultured HEp-2 cells. Cytotoxicity to HEp-2 cells was determined by measuring the amount of lactate dehydrogenase (LDH) released by HEp-2 calls when co-cultured with *C. freundii*. We first tested CF74 (aggregative adherence type) and CF72 (diffused adherence type) at 2, 6, 8, 10, 12, and 14 h. CF74 caused the release of more LDH than CF72. The difference was found to be statistically significant at 10 and 14 hours (*P*<0.01, Figure 4A). We then tested the remaining isolates at 10 hours. The released LDH levels averaged 7.45%, ranging from 4.5–24.3% (Table 1). Strain CF74, which showed aggregative adherence, also showed the highest cytotoxicity (Table 1). There was no evident trend of difference in cytotoxicity between STs and clusters. All strains except CF74 showed a rather small range of LDH release, from 4.5–8.4% with an average of 6.8%. This is similar to that induced by HB101 (5.4%). We concluded that all isolates except CF74 are likely to be non-cytotoxic.

The cytopathogenicity of *C. freundii* to cultured HEp-2 cells was also evident in cell morphology. HEp-2 cell monolayer detachment, rounding, and loss of viability were observed after 8 hours of growth, peaking after 12 hours of growth for strain CF74 (Figure 4B). Slight morphological changes in HEp-2 cells were observed for strain CF72. The EAggEC strain 17-2 and laboratory strain HB101 were used as positive and negative controls, respectively [21] (Figure 4B).

### Screening for known virulence genes

Previous reports show that some *C. freundii* strains carry Shiga toxins, heat-stable toxins, and cholera toxin homologs (*cfxAB*) [5], [9], [13]. We tested for the presence of Shiga toxin genes by PCR using primers reported previously, for heat stable and heat labile enterotoxins using primers from Rodas et al. and for *cfxAB* using primers designed based on sequences from *C. freundii* 09-1 [12], [13], [22], [23]. We also tested for the presence of *eae* gene although it has only been reported in *C. rodentium* [24]. None of the strains was found to contain the toxin genes or the *eae* gene tested. These results are not surprising considering that the prevalence of these toxin genes in *C. freundii* is likely to be low.

### Genome sequencing and comparison of two *C. freundii* strains (CF74 and CF72) with contrasting adherence and cytotoxicity phenotypes

#### Overall genome comparison

As described above, CF74 showed aggregative adherence and high cytotoxicity to HEp-2 cells, while CF72 showed weak adherence and relatively low cytotoxicity. In order to determine the genetic basis behind these differences, the genomes of *C. freundii* strains CF72 and CF74 were completely sequenced. The genomes of CF72 and CF74 each comprise a circular chromosome of 5,089,441 bp and 4,815,342 bp, encoding 5044 and 4600 genes including 13 and 10 pseudogenes, respectively. No plasmid was found in either strain. There are 87 tRNA genes, and eight rRNA operons in both strains. Many other enterobacteriaceae, including *C. rodentium*, contain only seven rRNA operons, but *C. freundii* has an extra copy. The two *C. freundii* genomes are co-linear, with one large inversion of approximately a third of the chromosome in CF74, as indicated by comparison to the *C. rodentium* genome ICC168, which is largely co-linear with CF72.

The two genomes were found to share 4260 genes. There were 778 genes unique to CF72 and 330 unique to CF74. Of these unique genes, 564 in CF72 and 210 in CF74 were found to be located on genomic islands. There were nine prophages, or phage remnants, in the CF72 genome and seven in CF74. Three of these prophages were shared by both strains. The larger size of the CF72 genome can partially account for the two extra prophages. Seven insertion sequence (IS) elements were found within the CF72 genome, but the copy number and distribution of IS in CF74 were not resolved due to the sequencing technology used.

An incomplete genome of *C. freundii* from strain ballerup 7851 was available for comparison [25]. Strain ballerup 7851 was isolated in 1939 from a female patient suffering from symptoms of gastroenteritis. This strain was found to express the Vi antigen of *Salmonella enterica* serovar Typhi [10]. The genome was previously sequenced using Roche 454 consisting of 357 contigs for a total of 4,904,659 by Petty et al. [25]. We mapped these contigs to our complete genome sequences and found that they shared 3269 genes. Ballerup 7851 is most similar to ST9, as indicated by the seven MLST genes. It is therefore only distantly related to CF72 and CF74. Whole-genome data were consistent with this observation. Note that because the ballerup 7851 genome is incomplete, there may be more shared genes in the ballerup 7851 genome than were discovered here. We did not determine the number of genes unique to ballerup 7851. The CF72 and CF74 genomes were also compared to that of *C. rodentium*, revealing 3026 shared genes with an average pairwise identity of 84.95% between *C. freundii* and *C. rodentium*.

#### Pathogenic islands and other virulence factors

We used the IslandPath program, which employs multiple methods to predict genomic islands (GIs) based on presence of mobility genes, tRNA genes, and genes that deviate significantly from the average GC content or show significant dinucleotide bias [26]. We found 42 Gis (Table S1). Fifteen of these were found to be shared by CF72 and CF74. The other 31 were found to carry strain-specific genes. Eighteen Gis were found to be specific to CF72 and thirteen to CF74 (Table S1). These 31 Gis encoded 774 strain-specific genes (69.9% of the total strain specific genes). Four pairs of Gis (GI18/GI31, GI20/GI36, GI26/GI42, and GI22/GI44) were found to have been inserted at the same sites of CF72 and CF74 but differ in gene contents. The preferred GI insertion sites were found to be mostly tRNA sites, each with 18 Gis flanked by a tRNA site. Seven genomic islands, six of which were found to encode fimbrial operons, are virulence-associated. Therefore, they are also pathogenic islands. Three Gis (GI35, GI38, and GI40) in CF74 are discussed in detail below. GI35 encodes a type VI secretion system (T6SS) which may be involved in secretion of cytotoxins, while GI38 and GI40 encode fimbriae, which may be associated with aggregative adherence.

We also searched for the presence of other secretion systems (T1SS to T5SS) and known virulence factors in the two *C. freudii* genomes. We found a putative T1SS (CF72_1264- CF72_1262 in CF72 and CF74_1113- CF74_1115 in CF74) but no other secretion systems. T1SS was found to secrete a range of substrates and may contribute to the difference in cytotoxicity between CF72 and CF74. However, because it is present in both strains, any contribution would have to involve the secretion of additional substrates from CF74 rather than the presence of T1SS per se. We also searched for T3SS effectors and found only a putative effector—methionine aminopeptidase—which showed 80% similarity to the homolog in *E. coli* O157:H7. Methionine aminopeptidase is present in both CF72 and CF74.

Autotransporters play an important role in adhesion and pathogenicity [27]. A putative autotransporter (CF74_1207) was found in CF74 only. This indicates that CF74_1207 may contribute to the differences in adhesion observed between CF74 and CF72. We screened for the presence of CF74_1207 in the other isolates. It was detected in seven isolates, including CF65, which showed similar level of adhesion to CF74. However, three CF74_1207-positive isolates showed little adhesion, which suggests that this gene does not play a large role in adhesion.

#### T6SS islands

**Figure 5:**
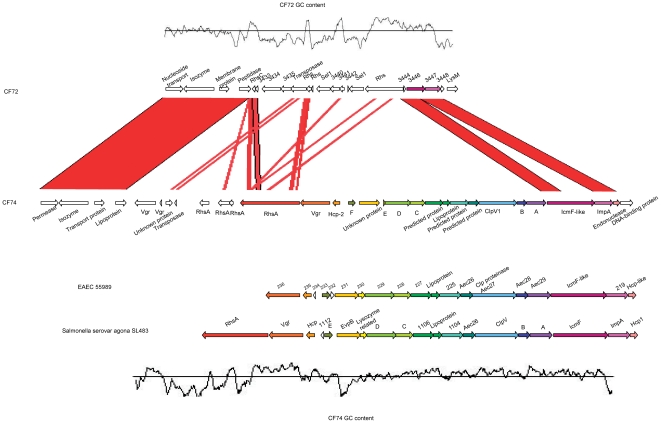
T6SS in *C. freundii* CF74. Here is shown a schematic representation of the *C. freundii* CF74 T6SS gene cluster compared to the T6SS clusters of *Salmonella enterica* serovar Agona strain SL483 and enteroaggregative *E. coli* (EAEC) strain 55989. CF72 has only two T6SS genes present at the same site where CF74 T6SS resides. Conserved T6SS components are highlighted in the same color. The GC content of CF74 T6SS gene clusters is shown at the bottom.

T6SS is present in many bacterial species and it is involved in virulence in several bacterial pathogens [28], [29]. Based on homologies to known T6SS, we identified a complete T6SS in CF74 and two T6SS genes in CF72. The CF74 T6SS gene cluster consists of 16 genes and appears to be a complete system, but in CF72 this region is replaced with non-homologous genes with only two T6SS genes remaining. The CF74 T6SS is most similar to that found in *S. enterica* serovar Agona SL483, showing 87.45% nucleotide sequence identity (Figure 5) [30]. Even the upstream *rhsA* element is conserved in *Salmonella*. However there are two extra *vgrG* homologs upstream of the CF74 T6SS, as discussed below. The *S. enterica* serovar Agona T6SS is located on SPI19 which is also present in several other *Salmonella* serovars including serovar Gallinarum, in which the T6SS contributes to colonization of chickens [31]. The *C. freundii* T6SS shares very low sequence homology with the functional T6SS (CST1) of *C. rodentium*. This shows that the T6SS systems in the two *Citrobacter* species do not share any recent common ancestry and were gained independently.

VgrG (valine-glycine repeats G) and Hcp (hemolysin-coregulated protein) are part of the T6SS machinery and are also secreted as effectors. There are three copies of *vgrG* and one *hcp* in the CF74 genome. One copy each of *vgrG* and *hcp* are located in the T6SS gene cluster and two additional copies of *vgrG* are located upstream of the T6SS gene cluster, separated by three *rhs* elements. The existence of multiple copies of *vgrG* in this strain is analogous to the existence of multiple copies in *Vibrio cholera*e and *Aeromonas hydrophila*, where 2 additional *vgrG* homologs were found outside the T6SS gene cluster [32], [33]. One of the VgrG proteins in *A. hydrophila*, VgrG1, was found to be an ADP ribosyltransferase with a vegetative insecticidal protein (VIP-2) domain at the carboxy-terminal end and induced host cell cytotoxicity by ADP ribosylation of host actin [34]. In *V. cholerae*, the VgrG1 C terminus shares an actin crosslinking domain (ACD) with the RtxA toxin, which mediates actin cross-linking and produces cell rounding [35]. However, none of the three VgrG homologs in CF74 contain a VIP-2 domain or an ACD. Despite this, we speculate that one or more of the three VgrG homologs in CF74 might have cytotoxic activity. VrgG proteins can have divergent C-termini with different functional domains like the VgrG1 proteins in *A. hydrophila* and *V. cholerae*.

We screened for the presence of T6SS in other strains using PCR, five genes from the T6SS gene cluster. Three genes were detected in seven strains (Table 1). CF74_0734 (*clpV1*) and CF74_0745 (*vgrG*1) were absent, suggesting that the T6SSs in these seven strains are non-functional and that only CF74 carries a functional T6SS. These results correlate with the fact that only CF74 is highly cytotoxic, providing further evidence that T6SS determines cytotoxicity.

#### Fimbriae islands

The two genomes were searched for fimbrial genes using BLAST searches against GenBank databases. Six regions were found to contain at least one gene similar to a fimbrial gene. These are here considered fimbriae islands. Five of these islands were also classified as genomic islands. However, one region uniquely present in CF72 and residing within GI26 was found to encode a fimbria with only two genes similar to fimbrial genes in other species: CF72_2817 encoding a PapD-like chaperone and CF72_2818 encoding a putative PapC-like usher protein. Among the five fimbrial genomic islands, two were uniquely present in CF74 and three in CF72.

**Figure 6:**
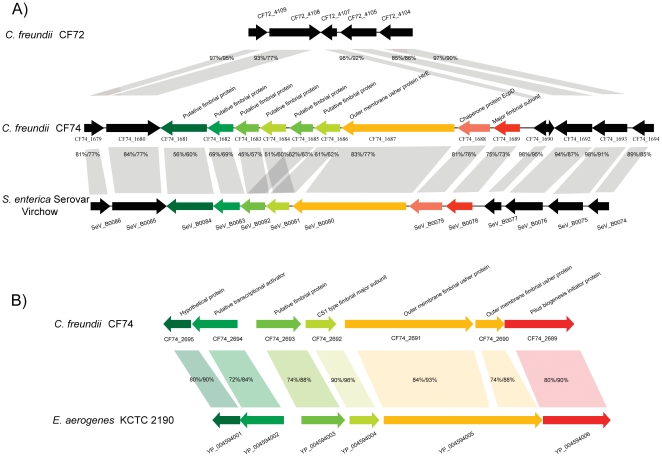
Genetic organization and comparison of fimbriae islands present in CF74. A. Genetic structure of GI38 and comparison to its homologous fimbrial gene cluster in *Salmonella enterica* serovar Virchow. The homologous region in CF72 is also shown. B. Genetic structure of GI40 and comparison with its homologous fimbrial gene cluster in *Enterobacter aerogenes*. Shown below gene bar are locus tags. Gene functions are shown at the top. The relative numbers of between two homologous genes and between the two strains are shown at the protein and nucleotide sequence levels.

The two fimbriae islands found in the CF74 genome are GI38 (CF74_1681 to CF74_1690) and GI40 (CF74_2689 to CF74_2695). GI38 consists of nine genes from CF74_1681 to CF74_1690. Based on the classification using the usher protein (CF74_1687) proposed by Baumler et al., this fimbria belongs to the γ1-related fimbrial clade which includes the well-known type 1 fimbriae and other fimbrial operons from a wide range of species belonging to *Enterobacteriaceae* [36]. The CF74 fimbrial operon is most similar to the *S. enterica* serovar Virchow *sth* operon with 45% to 83% identity at the amino acid level (Figure 6). GI40 encodes several proteins homologous to CS1 family fimbrial proteins. These include the CS1, CS2, CS4, and CFA/I pili of ETEC [37]. However, these genes are more homologous to an uncharacterized fimbrial gene cluster present in *Enterobacter aerogenes* KCTC 2190 genome, with an overall identity of 79% at the amino acid level (Figure 6), than to the ETEC fimbrial proteins (32% for the usher protein). The three fimbrial GIs uniquely present in CF72 are GI29, GI30, and GI31. GI29 (CF72 3877 to CF72_3881) is homologous to *lpf* operon encoding long polar fimbriae in *Salmonella* which plays an important role in adhesion to murine Peyer's patches [38]. GI30 is encoded by four genes (CF72 4084 to 4087). These genes are similar those that encode s-fimbriae (sfaA), which is an important adhesin for neonatal meningitis caused by *E. coli* [39]. This gene cluster was also found in the draft genome of a *Citrobacter* strain isolated from an IBD patient (GenBank accession no. NZ_ACDJ00000000). GI31, which includes seven genes (CF72 4097 to 4103), is most similar to genes encoding *sta* fimbriae in *Salmonella* [40].

Because fimbriae are widely known to be involved in adhesion, the fimbrial genes in CF72 and CF74 are likely to be involved in adhesion to HEp-2 cells [41]. However, it is difficult to infer whether the two fimbrial GIs present in CF74 rendered CF74 more adhesive. Further genetic characterization of these GIs is required to understand their precise contribution to adhesion.

#### Tellurite resistance islands

GI16 in CF72 encodes tellurite resistance (TeR). The genes are highly homologous to the tellurite genes in *E. coli* O157:H7, with DNA sequence identity ranging from 92% to 99%. In *E. coli* O157:H7, tellurite resistance is encoded by two duplicated *terrZABCDEF* gene clusters in O islands 43 and 48 in strain EDL933 and one gene cluster (SpLE1) in strain Sakai. These also harbor *iha*, encoding the adhesin and siderophore receptor Iha. The *iha* homolog in *C. freundii* is at a distant location and shares much less homology with that of other strains, only 73%.

We tested the other 25 *C. freundii* isolates for tellurite resistance and found four isolates (CF66, CF67, CF71, and CF78) in addition to CF72 to be resistant to potassium tellurite. We also determined the minimum inhibitory concentration (MIC) for each isolate. Four isolates (CF66, CF67, CF71, and CF72) showed an MIC of 128 µg/ml while one isolate (CF78) showed an MIC of 64 µg/ml. These five isolates all belonged to ST2. However, one of the ST2 isolates (CF94) was not tellurite resistant. It is likely that this isolate had lost the resistance due to excision of the *ter* gene cluster. The *ter* locus in O157:H7 has been found to be unstable with excision frequency as high as 1.81×10^−3^.

The virulence role of TeR in these *C. freundii* isolates is unclear. TeR is found in many pathogenic bacteria and sporadically present in different strains in *Enterobacteriaceae* [42]. Tellurite interacts with reduced thiols and TeR may be associated with oxidative stress resistance [42], [43], [44]. In *E. coli* O157:H7, deletion of the TeR gene cluster reduces adherence to HEp-2 cells *in vitro* [45]. However the TeR *C. freundii* isolates all show low adhesion and thus TeR is unlikely to have a role in adhesion in this case.

### Conclusion

No previous study has evaluated the genetic diversity of *C. freundii*. In this study, we analyzed isolates from diarrheal patients, animals, insects, and other sources. The majority of the isolated strains were found to carry the O157 O antigen. Isolates from animal sources shared the same STs as human isolates. These results suggest that these *C. freundii* strains may be widely present in human, animal, and other reservoirs. We did not attempt to sample commensal *C. freundii* isolates from healthy individuals. Future studies will address the genetic diversity of the *C. freundii* resident in intestinal tracts of humans and other animals.

In this study, we isolated a C. *freundii* strain, CF74, from an animal. This strain is cytotoxic and aggregative and therefore potentially pathogenic to humans. Aggregative adherence to epithelial cells has been recognized as a putative virulence factor contributing to bacterial pathogenicity. It was first observed in the 1980s in enteroaggregative *E. coli* and has now been observed in many species, including *Aeromonas*, *Klebsiella pneumoniae*, uropathogenic *Proteus mirabilis*, and atypical enteropathogenic *E. coli* [46], [47], [48], [49], [50], [51]. However, the public health importance of these aggregative bacterial pathogens was not appreciated until the so called Shiga-toxin-producing aggregative *E. coli* O104:H4 emerged in Germany. This resulted in approximately 25% of these infected patients developing HUS [52]. It was reported that the Shiga toxin 2 (*Stx*2) genes had been detected in *C. freundii* [12], [53]. These observations allow us to draw a possible parallel with the emergence of Shiga-toxin-producing aggregative *C. freundii*. A recent study by Pereira et al. reported that an aggregative *C. freundii* isolate obtained from a diarrheal patient concomitantly infected with EAEC synergistically enhanced adhesion of EAEC to HeLa cells *in vitro*, while a *C. freundii* isolate obtained from a healthy individual was only diffusely aggregative and showed no such synergy, demonstrating the indirect role of aggregative *C. freundii* in causing diarrhea [8].

We completely sequenced two *C. freundii* genomes. Comparative analysis of the two genomes revealed a core genome of 4260 genes and strain-specific genes of 564 and 210 for CF72 and CF74, respectively. We identified 42 GIs, with seven GIs related to virulence including fimbriae and a T6SS. The T6SS is carried only by CF74 which shares high similarity with the T6SS of *S. enterica* serovar Agona and is likely to be the key contributor to differences in cytotoxicity. Genome comparison revealed that strain CF74 acquired 13 GIs. Therefore, it seems that horizontal gene transfer played an important role in the emergence of cytotoxin-producing and aggregative *C. freundii*. Future studies will include functional characterization of these GIs to better understand the pathogenic potential of *C. freundii*.

## Materials and Methods

### Ethics statement

This study was reviewed and approved by the ethics committee of National Institute for Communicable Disease Control and Prevention, the Chinese CDC. All animals were treated in strict according to the guidelines for the Laboratory Animal Use and Care from Chinese CDC and the Rules for the Medical Laboratory Animal (1998) from Ministry of Health under the protocols approved by National Institute for Communicable Disease Control and Prevention. Human fecal specimens were acquired with the written informed consent of the diarrhea patients and with the approval of the ethics committee of National Institute for Communicable Disease Control and Prevention, according to the medical research regulations of Ministry of Health (permit number 2007-17-3).

### Strain isolation

The isolation of *C. freundii* was conducted during an enteric pathogen investigation in Henan Province (China). The procedure used was targeted for isolation of *E. coli* O157:H7, one of the pathogens routinely included in tests. Three to five microliters of fecal sample were transferred to 45 mL sterile mEC broth with Novobiocin (Oxoid, U.K.) and incubated at 36°C for 12 h to 14 h. After enrichment, 1.0 mL aliquots were processed by using Dynabeads anti-*E. coli* O157 (Dynal Biotech, Oslo, Norway), according to manufacturers' instructions. Bead and sample suspensions were incubated at room temperature for 30 min with continuous mixing on a Bellco roller drum (Bellco Glass, Inc., Vineland, NJ, U.S.) before plating onto CHROMagar O157 (CHROMagar, France). Mauve, white, and blue colonies from CHROMagar plates were further tested for agglutination with O157 antisera (China Center for Disease and Control) by standard methods and were further tested by API 20E (bioMerieux, France). No *E. coli* O157:H7 was isolated from any human person from which *C. freundii* was isolated.

None of the diarrheal patients who yielded *C. freundii* had any other known enteric bacterial pathogen in their feces. (We tested the samples for *Shigella*, *S. enterica*, and *V. cholerae*.) When *C. freundii* was isolated from a diarrheal patient, we attempted to isolate *C. freundii* from the patient's environment or nearby animals. All isolates were confirmed by 16 s RNA sequencing [54].

### Pulsed field gel electrophoresis analysis

Genomic DNA for PFGE was prepared in agarose plugs using the method and conditions previously established for *E. coli* O157:H7 [55]. It was found to be suitable for *C. freundii*. Slices of agarose plugs were digested with 50 U *Xba*I for 2 h, and electrophoresis was carried out in a 1% agarose SeaKem Gold gel with the CHEF DR III system (Bio-Rad, Hercules, California) with a switch time of 3–30 s and runtime of 18 h. The PFGE patterns were imported into Bionumerics (Applied Math, Belgium) for further analysis and manually edited for accuracy. Bands smaller than 20.5 kb were not included in the analysis. All patterns were visually inspected after computer analysis. Patterns indistinguishable by computer and visual inspection were assigned the same pattern designation. We assigned each PFGE pattern a unique identifier under the following naming conventions: CITX01.CNnnnn, with CIT for *Citrobacter*, X01 for *Xba* I, CN for China, and nnnn for a sequential number.

### Multi-locus sequence typing (MLST)

The seven housekeeping genes used for MLST were *aspC*, *clpX*, *fadD*, *mdh*, *arcA*, *dnaG* and *lysP*, based on the *E. coli* MLST scheme developed by Whittam and colleagues [46]. The MLST primers (Table 2) were designed based on our draft genome sequence of a *C. freundii* isolate (unpublished). They were synthesized commercially by Shanghai Sangon Biological Engineering Technology and Services (China).

PCR products were verified on 1% agarose gels and purified for sequencing. This was performed commercially done at Shanghai Sangon Biological Engineering Technology and Services (China) using ABI dye terminator sequencing. DNA molecules were sequenced in both directions. Sequences were edited using SeqMan7.0.

### HEp-2 cell adhesion assay

The human epidermoid laryngocarcinoma (HEp-2, CCC0068) cell line was obtained from the cell resource center at Peking Union Medical College. The cells were maintained in 25 cm^2^ flasks with RPMI-1640 medium (Gibco) supplemented with 10% fetal bovine serum (Gibco), at 37°C in 5% CO_2_. Adhesion experiments on the *C. freundii* isolates were performed following the method described by Mange et al. [19]. HEp-2 cells were seeded onto glass coverslips (12 mm diameter) in 24-well plates at a density of 5×10^4^ cells per well, 24 h before infection. The medium was replaced before infection with RPMI-1640 medium containing 1% mannose. A mixture with an MOI (MOI; number of bacteria per number of mammalian cells) of 100∶1 was added to the HEp-2 monolayers in a 1 ml culture medium. Bacteria were allowed to adhere for 180 min at 37°C in 5% CO_2_. Enteroaggregative *E. coli* (EAggEC) strain 042 was used for aggregative adherence and enteropathogenic *E. coli* (EPEC) strain 2348/69 was used for localized adherence. These were included for comparison of different types of adhesion, and *E. coli* strain HB101 was included as a non-adhering control. After incubation, each well was rinsed five times with phosphate-buffered saline (PBS), cells were fixed by pre-cooled methanol (−20°C) for 15 min, stained with 1% Giemsa stain (Sigma-Aldrich), and examined under a light transmission microscope at a magnification of ×1,000 (oil immersion). An adhesion index (<1; >1 and <50; >50) describing the mean number of bacteria per HEp-2 after examination of 10 visual fields per coverslip was determined [19]. Infections were repeated three times in duplicate.

### HEp-2 cell cytotoxicity assay

HEp-2 cells (5×10^4^ cells/well) were seeded in 96-well tissue culture plates (Corning) with RPMI-1640 medium (Gibco) supplemented with 5% fetal bovine serum (Gibco), and infected as described above. After infection lasting different amounts of time, the cell monolayers were washed with PBS (pH 7.4) and stained with a crystal violet solution (1.3% crystal violet and 5% ethanol in PBS), and examined under a light transmission microscope at a magnification of ×100. Washed bacterial cultures were added to the HEp-2 cells, and the dishes were incubated at 37°C in 5% CO_2_ for up to 12 hours. A positive result was indicated by rounding up and detachment of monolayer HEp-2 cells from the culture dishes. EAEC 17-2 were used as positive controls, *E. coli* strain HB101 was included as a non-cytotoxic control. All experiments were performed three times in duplicate.

The lactate dehydrogenase (LDH) released by HEp-2 cells was determined using the Cytotox96 kit (Promega) according to the manufacturer's instructions. Lysed cells and cells with HB101 were used as positive and negative controls, respectively. The relative amount of cytotoxicity was expressed as (experimental release-spontaneous release)/(maximum release -spontaneous release)×100, in which the spontaneous release was the amount of LDH activity in the supernatant of uninfected cells and the maximum release was that when cells were lysed with the lysis buffer provided by the manufacturer. *E. coli* strain HB101 was included as a non-cytotoxic control. All experiments were performed three times in duplicate [56].

### Detection of virulence genes and other genes by PCR

The primers used for detection of virulence genes, O157 O antigen genes and the *ter* genes encoding tellurite resistance were listed in Table S2. The *ter* gene primers were based on a draft *C. freundii* genome sequence (unpublished). Each PCR reaction included 2.5 µl of DNA template (approx. 20 ng), 0.5 µl (30 pmol/µl) of each forward and reverse primers, 0.5 µl 10 mM dNTPs, 5 µl 10× PCR buffer (500 mM KCl, 100 mM Tris-HCl pH 9.0, 1% Triton® X-100 and 15 mM MgCl_2_), 0.25 µl (1.25 U) Taq polymerase (Takara) and MilliQ water to a total volume of 50 µl. PCR cycles were performed in a Ferotec Thermal Cycler TC-48/T/H(a) under the following conditions: initial DNA denaturation for 5 min at 94°C; followed by DNA denaturation for 60 sec at 94°C, primer annealing for 30 s at appropriate temperatures for each given primer pair shown in Table S2 and polymerization for 30–120 s at 72°C for 30 cycles with a final extension of 10 min at 72°C. PCR products were verified on ethidium bromide stained agarose gels.

### Genome sequencing and bioinformatic analysis

Chromosomal DNA from *C. freundii* strain CF72 was isolated with standard protocols. Two libraries (inserts of 2–3 kb and 6–8 kb) were generated by mechanical shearing of chromosomal DNA. Double-ended plasmid sequencing reactions were performed using an ABI BigDye Terminator V3.1 Cycle Sequencing Kit and an ABI 3730 Automated DNA Analyzer (Applied Biosystems). In this way, 68,886 reads were generated and assembled using the PHRED PHRAP and CONSED (31) programs. Linkages among contigs were based on the sequences of gap-spanning clones. Sequence gaps were closed by primer walking on linking clones or sequencing PCR products amplified from genomic DNA. All repeated and low-quality DNA regions were verified by PCR and sequencing of the protein product. The ribosomal RNA operon sequences were assembled separately by construction of DNase I shotgun banks. The final genome was assembled as one contig based on 70,827 reads.

Whole-genome sequencing of *C. freundii* 74 was performed by combining GS FLX and Solexa paired-end sequencing technologies. Genomic libraries containing 8 kb inserts were constructed and 152,636 reads (65.3% paired-end) were produced using the GS FLX system, giving 10.76-fold coverage of the genome. About 97.86% of reads were assembled into 12 large scaffolds using Newbler (454 Life Sciences, Branford, CT, U.S.). A total of 18,847,864 reads (inserts of 450 bp and 3 kb) were generated using an Illumina Solexa Genome Analyzer IIx and were mapped to the scaffolds using the Burrows-Wheeler Alignment (BWA) tool. Relationships between scaffolds were resolved by multiplex PCR. All gaps were filled by local assembly of the Solexa/Roche 454 reads or sequencing PCR products using an ABI 3730 capillary sequencer. To avoid sequence miscalls by Roche/454 FLX in homopolymers, all questionable sites were checked by the coverage of 454 reads. Gaps were closed by directed PCR and sequencing with BigDye terminator chemistry on an ABI 3730 capillary sequencer. However, the copy number and distribution of IS sequences were not resolved. Open reading frames 30 amino acids in length and longer were predicted using Glimmer 3.0 and verified manually. Transfer RNA and ribosomal RNA genes were predicted using tRNAscan-SE and by similarity to the rRNA genes in public databases, respectively [57]. Artemis was used to collate data and facilitate annotation [58]. Function predictions were based on BLASTP similarity searches against the GenBank protein database, Swiss-Prot protein database and the clusters of orthologous groups (COG) database. Genome annotation and comparison were done as described previously [59]. The co-linear blocks of the genomes were determined using BLASTN. Then the alignment within each of the block was obtained using Mauve [60].

Detection of genomic islands was performed using IslandPath which employs multiple methods to predict genomic islands (GIs) based on the presence of mobility genes, tRNA genes and genes that have significant deviations from the average GC content or dinucleotide bias [24]. The putative islands identified were then manually checked for island features and island boundaries.

### Determination of tellurite minimum inhibitory concentration

Tellurite MICs were determined as described by Taylor et al. [61]. Cultures were grown overnight in LB (Oxoid, U.K.). One hundred microliters of broth culture was diluted in 5 ml of 1× PBS, and 10 µl of this dilution was plated on LB agar (Oxoid, U.K.) plates containing increasing twofold concentrations of potassium tellurite (Fluka) ranging from two to 1,024 µg/ml. The plates were incubated at 37°C overnight. The MIC was defined as the lowest concentration of tellurite capable of completely inhibiting bacterial growth.

## Supporting Information

Table S1 Shared and strain-specific genomic islands of *C. freundii* CF72 and CF74.
(DOCX)

Table S2 PCR primers used in this study.
(DOCX)

## References

[1] Kaur P, Chakraborti A, Asea A (2010). Enteroaggregative *Escherichia coli:* An Emerging Enteric Food Borne Pathogen.. Interdiscip Perspect Infect Dis.

[2] Okeke IN, Ojo O, Lamikanra A, Kaper JB (2003). Etiology of acute diarrhea in adults in southwestern Nigeria.. J Clin Microbiol.

[3] Scavia G, Staffolani M, Fisichella S, Striano G, Colletta S (2008). Enteroaggregative *Escherichia coli* associated with a foodborne outbreak of gastroenteritis.. J Med Microbiol.

[4] Guerrant RL, Dickens MD, Wenzel RP, Kapikian AZ (1976). Toxigenic bacterial diarrhea: nursery outbreak involving multiple bacterial strains.. J Pediatr.

[5] Tschape H, Prager R, Streckel W, Fruth A, Tietze E (1995). Verotoxinogenic *Citrobacter freundii* associated with severe gastroenteritis and cases of haemolytic uraemic syndrome in a nursery school: green butter as the infection source.. Epidemiol Infect.

[6] Warner RD, Carr RW, McCleskey FK, Johnson PC, Elmer LM (1991). A large nontypical outbreak of Norwalk virus. Gastroenteritis associated with exposing celery to nonpotable water and with *Citrobacter freundii*.. Arch Intern Med.

[7] Parida SN, Verma IC, Deb M, Bhujwala RA (1980). An outbreak of diarrhea due to *Citrobacter freundii in* a neonatal special care nursery.. Indian J Pediatr.

[8] Pereira AL, Silva TN, Gomes AC, Araujo AC, Giugliano LG (2010). Diarrhea-associated biofilm formed by enteroaggregative *Escherichia coli* and aggregative *Citrobacter freundii*: a consortium mediated by putative F pili.. BMC Microbiol.

[9] Guarino A, Capano G, Malamisura B, Alessio M, Guandalini S (1987). Production of *Escherichia coli* STa-like heat-stable enterotoxin by *Citrobacter freundii* isolated from humans.. J Clin Microbiol.

[10] Kauffmann F, Moller E (1940). A new type of *Salmonella (S. ballerup)* with Vi-antigen.. JHyg(Lond).

[11] Guarino A, Giannella R, Thompson MR (1989). *Citrobacter freundii* produces an 18-amino-acid heat-stable enterotoxin identical to the 18-amino-acid *Escherichia coli* heat-stable enterotoxin (ST Ia).. Infect Immun.

[12] Schmidt H, Montag M, Bockemuhl J, Heesemann J, Karch H (1993). Shiga-like toxin II-related cytotoxins in *Citrobacter freundii* strains from humans and beef samples.. Infect Immun.

[13] Karasawa T, Ito H, Tsukamoto T, Yamasaki S, Kurazono H (2002). Cloning and characterization of genes encoding homologues of the B subunit of cholera toxin and the *Escherichia coli* heat-labile enterotoxin from clinical isolates of *Citrobacter freundii* and *E. coli*.. Infect Immun.

[14] Mundy R, MacDonald TT, Dougan G, Frankel G, Wiles S (2005). *Citrobacter rodentium* of mice and man.. Cell Microbiol.

[15] Struelens MJ (1996). Consensus guidelines for appropriate use and evaluation of microbial epidemiologic typing systems.. Clinical Microbiology and Infection.

[16] Kobayashi M, Sasaki T, Saito N, Tamura K, Suzuki K (1999). Houseflies: not simple mechanical vectors of enterohemorrhagic *Escherichia coli* O157:H7.. Am J Trop Med Hyg.

[17] Lacher DW, Steinsland H, Blank TE, Donnenberg MS, Whittam TS (2007). Molecular evolution of typical enteropathogenic *Escherichia coli*: clonal analysis by multilocus sequence typing and virulence gene allelic profiling.. Journal of Bacteriology.

[18] Feil EJ, Li BC, Aanensen DM, Hanage WP, Spratt BG (2004). eBURST: inferring patterns of evolutionary descent among clusters of related bacterial genotypes from multilocus sequence typing data.. Journal of Bacteriology.

[19] Mange JP, Stephan R, Borel N, Wild P, Kim KS (2006). Adhesive properties of *Enterobacter sakazakii* to human epithelial and brain microvascular endothelial cells.. BMC Microbiol.

[20] Yamamoto T, Koyama Y, Matsumoto M, Sonoda E, Nakayama S (1992). Localized, aggregative, and diffuse adherence to HeLa cells, plastic, and human small intestines by *Escherichia coli* isolated from patients with diarrhea.. J Infect Dis.

[21] Veilleux S, Holt N, Schultz BD, Dubreuil JD (2008). *Escherichia coli* EAST1 toxin toxicity of variants 17-2 and O 42.. Comp Immunol Microbiol InfectDis.

[22] Zhang W, Bielaszewska M, Kuczius T, Karch H (2002). Identification, characterization, and distribution of a Shiga toxin 1 gene variant (stx(1c)) in *Escherichia coli* strains isolated from humans.. J Clin Microbiol.

[23] Rodas C, Iniguez V, Qadri F, Wiklund G, Svennerholm AM (2009). Development of multiplex PCR assays for detection of enterotoxigenic *Escherichia coli* colonization factors and toxins.. J Clin Microbiol.

[24] Hubbard AL, Harrison DJ, Moyes C, McOrist S (1998). Direct detection of eae-positive bacteria in human and veterinary colorectal specimens by PCR.. J Clin Microbiol.

[25] Petty NK, Bulgin R, Crepin VF, Cerdeno-Tarraga AM, Schroeder GN (2010). The *Citrobacter rodentium* genome sequence reveals convergent evolution with human pathogenic *Escherichia coli*.. J Bacteriol.

[26] Hsiao W, Wan I, Jones SJ, Brinkman FS (2003). IslandPath: aiding detection of genomic islands in prokaryotes.. Bioinformatics.

[27] Navarro-Garcia F, Elias WP (2011). Autotransporters and virulence of enteroaggregative *E. coli*.. Gut Microbes.

[28] Dudley EG, Thomson NR, Parkhill J, Morin NP, Nataro JP (2006). Proteomic and microarray characterization of the AggR regulon identifies a pheU pathogenicity island in enteroaggregative *Escherichia coli*.. Mol Microbiol.

[29] Schwarz S, Hood RD, Mougous JD (2010). What is type VI secretion doing in all those bugs?. Trends Microbiol.

[30] Blondel CJ, Jimenez JC, Contreras I, Santiviago CA (2009). Comparative genomic analysis uncovers 3 novel loci encoding type six secretion systems differentially distributed in *Salmonella serotypes*.. BMC Genomics.

[31] Blondel CJ, Yang HJ, Castro B, Chiang S, Toro CS (2010). Contribution of the type VI secretion system encoded in SPI-19 to chicken colonization by *Salmonella enterica* serotypes Gallinarum and Enteritidis.. PLoS One.

[32] Pukatzki S, Ma AT, Sturtevant D, Krastins B, Sarracino D (2006). Identification of a conserved bacterial protein secretion system in *Vibrio cholerae* using the Dictyostelium host model system.. Proc Natl Acad Sci USA.

[33] Suarez G, Sierra JC, Sha J, Wang S, Erova TE (2008). Molecular characterization of a functional type VI secretion system from a clinical isolate of *Aeromonas hydrophila*.. Microb Pathog.

[34] Suarez G, Sierra JC, Erova TE, Sha J, Horneman AJ (2010). A type VI secretion system effector protein, VgrG1, from *Aeromonas hydrophila* that induces host cell toxicity by ADP ribosylation of actin.. J Bacteriol.

[35] Sheahan KL, Cordero CL, Satchell KJ (2004). Identification of a domain within the multifunctional *Vibrio cholerae* RTX toxin that covalently cross-links actin.. Proc Natl Acad Sci U S A.

[36] Nuccio SP, Baumler AJ (2007). Evolution of the chaperone/usher assembly pathway: fimbrial classification goes Greek.. Microbiol Mol Biol Rev.

[37] Anantha RP, McVeigh AL, Lee LH, Agnew MK, Cassels FJ (2004). Evolutionary and functional relationships of colonization factor antigen I and other class 5 adhesive fimbriae of enterotoxigenic *Escherichia coli*.. Infect Immun.

[38] Baumler AJ, Tsolis RM, Heffron F (1996). The lpf fimbrial operon mediates adhesion of *Salmonella typhimurium* to murine Peyer's patches.. Proc Natl Acad Sci USA.

[39] Saukkonen KM, Nowicki B, Leinonen M (1988). Role of type 1 and S fimbriae in the pathogenesis of *Escherichia coli* O18:K1 bacteremia and meningitis in the infant rat.. Infect Immun.

[40] Townsend SM, Kramer NE, Edwards R, Baker S, Hamlin N (2001). *Salmonella enterica* serovar Typhi possesses a unique repertoire of fimbrial gene sequences.. Infect Immun.

[41] Wagner C, Hensel M (2011). Adhesive mechanisms of *Salmonella enterica*.. Adv Exp Med Biol.

[42] Taylor DE (1999). Bacterial tellurite resistance.. Trends Microbiol.

[43] Turner RJ, Weiner JH, Taylor DE (1999). Tellurite-mediated thiol oxidation in *Escherichia coli*.. Microbiology.

[44] Valková d, Valkovičová L, Vávrová S, Kováčová E, Mravec J (2007). The contribution of tellurite resistance genes to the fitness of *Escherichia coli* uropathogenic strains.. Central European Journal of Biology.

[45] Yin X, Wheatcroft R, Chambers JR, Liu B, Zhu J (2009). Contributions of O island 48 to adherence of enterohemorrhagic *Escherichia coli* O157:H7 to epithelial cells in vitro and in ligated pig ileal loops.. Appl Environ Microbiol.

[46] Vial PA, Robins-Browne R, Lior H, Prado V, Kaper JB (1988). Characterization of enteroadherent-aggregative *Escherichia coli*, a putative agent of diarrheal disease.. J Infect Dis.

[47] Barros SF, Abe CM, Rocha SP, Ruiz RM, Beutin L (2008). *Escherichia coli* O125ac:H6 encompasses atypical enteropathogenic *E. coli* strains that display the aggregative adherence pattern.. J Clin Microbiol.

[48] Favre-Bonte S, Darfeuille-Michaud A, Forestier C (1995). Aggregative adherence of *Klebsiella pneumoniae* to human intestine-407 cells.. Infect Immun.

[49] Neves MS, Nunes MP, Milhomem AM (1994). *Aeromonas species* exhibit aggregative adherence to HEp-2 cells.. J Clin Microbiol.

[50] Nucleo E, Fugazza G, Migliavacca R, Spalla M, Comelli M (2010). Differences in biofilm formation and aggregative adherence between beta-lactam susceptible and beta-lactamases producing P. mirabilis clinical isolates.. New Microbiol.

[51] Rocha SP, Elias WP, Cianciarullo AM, Menezes MA, Nara JM (2007). Aggregative adherence of uropathogenic *Proteus mirabilis* to cultured epithelial cells.. FEMS Immunol Med Microbiol.

[52] Frank C, Werber D, Cramer JP, Askar M, Faber M (2011). Epidemic Profile of Shiga-Toxin-Producing *Escherichia coli* O104:H4 Outbreak in Germany - Preliminary Report.. N Engl J Med.

[53] Herold S, Karch H, Schmidt H (2004). Shiga toxin-encoding bacteriophages–genomes in motion.. Int J Med Microbiol.

[54] Baker GC, Smith JJ, Cowan DA (2003). Review and re-analysis of domain-specific 16S primers.. J Microbiol Methods.

[55] Ribot EM, Fair MA, Gautom R, Cameron DN, Hunter SB (2006). Standardization of pulsed-field gel electrophoresis protocols for the subtyping of *Escherichia coli* O157:H7, *Salmonella*, and *Shigella* for PulseNet.. Foodborne Pathog Dis.

[56] Roberts PH, Davis KC, Garstka WR, Bhunia AK (2001). Lactate dehydrogenase release assay from Vero cells to distinguish verotoxin producing *Escherichia coli* from non-verotoxin producing strains.. J Microbiol Methods.

[57] Lowe TM, Eddy SR (1997). tRNAscan-SE: a program for improved detection of transfer RNA genes in genomic sequence.. Nucleic Acids Res.

[58] Carver T, Berriman M, Tivey A, Patel C, Bohme U (2008). Artemis and ACT: viewing, annotating and comparing sequences stored in a relational database.. Bioinformatics.

[59] Feng L, Reeves PR, Lan R, Ren Y, Gao C (2008). A recalibrated molecular clock and independent origins for the cholera pandemic clones.. PLoS One.

[60] Darling AC, Mau B, Blattner FR, Perna NT (2004). Mauve: multiple alignment of conserved genomic sequence with rearrangements.. Genome Res.

[61] Taylor DE, Rooker M, Keelan M, Ng LK, Martin I (2002). Genomic variability of O islands encoding tellurite resistance in enterohemorrhagic *Escherichia coli* O157:H7 isolates.. J Bacteriol.

