# Supplementary material for: Isolation and Characterization of Cytotoxic, Aggregative Citrobacter freundii
Source: PLoS One. 2012 Mar 21;7(3):e33054. doi: 10.1371/journal.pone.0033054 (PMC3310003; doi:10.1371/journal.pone.0033054)
Supplement: Table S1 — Shared and strain-specific genomic islands of C. freundii CF72 and CF74. (DOCX) [file pone.0033054.s001.docx]

Table S1 Shared and strain-specific genomic islands of *C. freundii* CF72 and CF74

| Genomic  island | Strain specific | Start locus  tag | End locus  tag | Length (bp) | Putative  functions |
| --- | --- | --- | --- | --- | --- |
| 1 | Both | CF72_0223 | CF72_0242 | 23,034 | Metabolism |
| 2 | Both | CF72_0295 | CF72_0312 | 15,416 | Unknown |
| 3 | Both | CF72_0395 | CF72_0406 | 12,462 | Metabolism |
| 4 | Both | CF72_0586 | CF72_0596 | 14,801 | Transcription |
| 5 | Both | CF72_0855 | CF72_0868 | 16,233 | Metabolism |
| 6 | Both | CF72_1014 | CF72_1048 | 52,459 | Transport |
| 7 | Both | CF72_1214 | CF72_1262 | 35,931 | Phage related |
| 8 | Both | CF72_1456 | CF72_1479 | 26,118 | Metabolism |
| 9 | Both | CF72_1669 | CF72_1680 | 14,505 | Drug resistance |
| 10 | Both | CF72_1778 | CF72_1786 | 8,242 | Unknown |
| 11 | Both | CF72_1856 | CF72_1878 | 23,997 | O-antigen |
| 12 | Both | CF72_2722 | CF72_2730 | 7,871 | Multidrug resistance |
| 13 | Both | CF72_2956 | CF72_2966 | 11,079 | ABC transport |
| 14 | Both | CF72_3477 | CF72_3482 | 5,212 | TolQRA bile resistance |
| 15 | Both | CF72_5010 | CF72_5028 | 17,616 | ATP syntheses |
| 16 | CF72-specific | CF72_0070 | CF72_0113 | 35,792 | Tellurite resistance |
| 17 | CF72-specific | CF72_0410 | CF72_0422 | 11,560 | Malonate metabolism |
| 18 | CF72-specific | CF72_0693 | CF72_0711 | 14,912 | Phage related |
| 19 | CF72-specific | CF72_0797 | CF72_0828 | 30,565 | Metabolism |
| 20 | CF72-specific | CF72_1186 | CF72_1213 | 25,722 | Arsenical pump |
| 21 | CF72-specific | CF72_1289 | CF72_1327 | 29,309 | Phage related |
| 22 | CF72-specific | CF72_1952 | CF72_2005 | 39,275 | Phage related |
| 23 | CF72-specific | CF72_2109 | CF72_2171 | 40,821 | Phage related |
| 24 | CF72-specific | CF72_2351 | CF72_2415 | 49,831 | Phage related |
| 25 | CF72-specific | CF72_2521 | CF72_2531 | 6,777 | Hypothetical protein |
| 26 | CF72-specific | CF72_2816 | CF72_2835 | 17,646 | Fimbriae and arsenical resistance |
| 27 | CF72-specific | CF72_3018 | CF72_3082 | 46,796 | Phage related |
| 28 | CF72-specific | CF72_3720 | CF72_3743 | 26,387 | Metabolism |
| 29 | CF72-specific | CF72_3877 | CF72_3881 | 5,312 | Fimbriae |
| 30 | CF72-specific | CF72_4084 | CF72_4088 | 5,264 | Fimbriae |
| 31 | CF72-specific | CF72_4097 | CF72_4103 | 7,206 | Fimbriae |
| 32 | CF72-specific | CF72_4369 | CF72_4390 | 27,248 | Phage related |
| 33 | CF72-specific | CF72_4528 | CF72_4614 | 80,848 | Phage related |
| 34 | CF74-specific | CF74_0635 | CF74_0641 | 5,548 | Phage related |
| 35 | CF74-specific | CF74_0726 | CF74_0760 | 49,073 | T6SS |
| 36 | CF74-specific | CF74_1104 | CF74_1112 | 8,409 | Phage related |
| 37 | CF74-specific | CF74_1425 | CF74_1440 | 21,944 | DnD system |
| 38 | CF74-specific | CF74_1681 | CF74_1689 | 8,231 | Fimbriae |
| 39 | CF74-specific | CF74_1957 | CF74_1973 | 26,503 | Type I R-M system |
| 40 | CF74-specific | CF74_2689 | CF74_2695 | 6,811 | Fimbriae |
| 41 | CF74-specific | CF74_2983 | CF74_2995 | 12,519 | Phage related |
| 42 | CF74-specific | CF74_3085 | CF74_3094 | 7,591 | Drug resistance |
| 43 | CF74-specific | CF74_3703 | CF74_3739 | 33,886 | Phage related |
| 44 | CF74-specific | CF74_3789 | CF74_3841 | 43,561 | Phage related |
| 45 | CF74-specific | CF74_4235 | CF74_4242 | 11,455 | Phage related |
| 46 | CF74-specific | CF74_4258 | CF74_4279 | 22,577 | Transport |
