# Supplementary material for: Isolation and Characterization of Cytotoxic, Aggregative Citrobacter freundii
Source: PLoS One. 2012 Mar 21;7(3):e33054. doi: 10.1371/journal.pone.0033054 (PMC3310003; doi:10.1371/journal.pone.0033054)
Supplement: Table S2 — PCR primers used in this study. (DOCX) [file pone.0033054.s002.docx]

Table S2 PCR primers used in this study

| Primer | Sequence(5'-3') | Annealing temperature | Reference |
| --- | --- | --- | --- |
| *eae* |  |  | (24) |
| p1 | ATTATGGAACGGCAGAGG | 55 |  |
| p2 | GGAAGGAAAAAACGCTGAC |  |  |
| p3 | GAGTGGTAATAACTTTGACGG | 50 |  |
| p4 | GTAAAGCGGGAGTCAATG |  |  |
| P11e | GAGGAAGGTGGGGATGACG | 55 |  |
| P13b | AGGCCCGGGAACGTATTCAC |  |  |
| *stx* |  |  | (12,22) |
| MK1 | TTTACGATAGACTTCTCGAC | 43 |  |
| MK2 | CACATATAAATTATTTCGCTC |  |  |
| KS7 | TGAAAAAAACATTATTAATAGC | 50 |  |
| KS8 | GCTATTCTGAGTCAACG |  |  |
| GK1 | TGTATATTATTTAAATGG | 52 |  |
| GK2 | TATTTACCCGTTGTATATAAAAA |  |  |
| GK3 | AAGATGTTTATGGCG | 43 |  |
| GK4 | CAGTCATTATTAAACTGCAC |  |  |
| GK1 | TGTATATTATTTAAATGG | 52 |  |
| GK4 | CAGTCATTATTAAACTGCAC |  |  |
| *LT/ST* |  |  | (23) |
| LT-F | ACGGCGTTACTATCCTCTC | 52 |  |
| LT-R | TGGTCTCGGTCAGATATGTG |  |  |
| STh-F | TTCACCTTTCCCTCAGGATG | 52 |  |
| STh-R | CTATTCATGCTTTCAGGACCA |  |  |
| *cfxAB* |  |  | this study |
| cfxa-F | AGCTGAGAGGACCCCAAACGA | 55 |  |
| cfxa-R | ACTCGGTAACCGGCCGTCCT |  |  |
| cfxb-F | CGCTGTATTCTAAAGCGGCACCT | 55 |  |
| cfxb-R | TTCCAGACACAAAGCTTGCTGACTT |  |  |
| *O157* |  |  |  |
| gndF | GCTGTGATGGGGCGCAACCT | 55 |  |
| gndR | GCGCACGAATGATGCAGCCCGC |  |  |
| manBF | GGACCAGTTCGCCCAGCGAC | 60 |  |
| manBR | CGCCTGGCGGAAGCCAATGA |  |  |
| manCF | CTGGTGCTGGCGGCAGATCA | 59 |  |
| manCR | TGAGCGATGATGGTGCATGCGT |  |  |
| gmmF | GAGCGCTTGACGTTGGCGGA | 58 |  |
| gmmR | AGGCAACTCTCCTGTCCCCGA |  |  |
| fclF | ATGGTGGGGACCGCCATCGT | 60 |  |
| fclR | TCCCGCTTCCCCACACCACA |  |  |
| gmdF | TCCCCAATTGCGCGGCAGAC | 60 |  |
| gmdR | AGATGCTATGGGGACATTGCGCC |  |  |
| wbdPF | TCCCGAGAGCTACCGCATCA | 57 |  |
| wbdPR | TGGGGAGGCGGGGTCGATTT |  |  |
| perF | AGGGGGATGAGGTCATCGTTCCA | 58 |  |
| perR | GCCAGCCCTATTGCCGCACA |  |  |
| wzxF | GCGCTGCGCTCAAACTTGCT | 58 |  |
| wzxR | TGGACGGCAGCAGGAAAGGT |  |  |
| wbdOF | ACGATGCAATGAATAAAGGGCACAA | 56 |  |
| wbdOR | GGGAGGCTAACCCCTCCGGA |  |  |
| wzyF | ACCACCTTTCTTAGCATGCTGTGC | 57 |  |
| wzyR | TCCGGGAGGGGCGAAACTGA |  |  |
| wbdNF | TCCACGTCCATTGTGCTGCT | 56 |  |
| wbdNR | ACAAAGGCGTCGCGGAAAGT |  |  |
| galFF | CACCTGGTTGGGTATGCT | 53 |  |
| galFR | CGGGCGTGACTATTATGAA |  |  |
| *ter* |  |  | this study |
| terAF | GCTCACATACCGGGATCG | 56 |  |
| terAR | CTGGCGGGTCAGTTCGTC |  |  |
| terBF | TGCCAGACTTGTAAACGA | 52 |  |
| terBR | CCAGAATACGGACCAGAG |  |  |
| terCF | GTATTGCTGTTGCGAAGA | 53 |  |
| terCR | GCATCAAGGTCAAAGTCC |  |  |
| terDF | CCTGTATGTTGTATTGGGTG | 53 |  |
| terDR | TGGTTGGTGCTTCTTTGG |  |  |
| terEF | CGCAACAACAGTGAGTGG | 53 |  |
| terER | TCCCGAATATCAGGAACG |  |  |
| terFF | AGCAGGACAGCATTGAGA | 52 |  |
| terFR | AAGGAGTCGTTCAGGGTC |  |  |
| terXF | ACGTGCCTCATTGTCATA | 52 |  |
| terXR | GTGCGAGCATTTCGGTAG |  |  |
| terWF | TTTGTAGGCAGAAAGGAG | 54 |  |
| terWR | TTTGAGGGCATAGAACAC |  |  |
| terZF | TTGGAGCATCTTTTATTCG | 53 |  |
| terZR | CACTGACCAGGCTGACGG |  |  |
| *T6SS* |  |  | this study |
| CF74_0731F | CGGCTTTCACCGGCGAGGTT |  |  |
| CF74_0731R | ACGGATGTGCTGCACCGTCG |  |  |
| CF74_0734F | TGCGGCAAATCGGTGATGACCG |  |  |
| CF74_0734R | GCCGCAATAGCTGGCGATGAAAG |  |  |
| CF74_0737F | ACCAGCAGGCGGTAGTGGTGTC |  |  |
| CF74_0737R | AGGGCGTGGTTTCCTTGCCTGG |  |  |
| CF74_0743F | GGGGCAAACCCAGGGCAATATCACC |  |  |
| CF74_0743R | GCCAGTCATCAGCACCAGAGGTGC |  |  |
| CF74_0745F | ACGAGGAACATGCCCTCACCAGC |  |  |
| CF74_0745R | TCGTCATTACCAGCCGGGCCT |  |  |
